# Aberrant Brain Function in Active-Stage Ulcerative Colitis Patients: A Resting-State Functional MRI Study

**DOI:** 10.3389/fnhum.2019.00107

**Published:** 2019-04-03

**Authors:** Weijie Fan, Si Zhang, Junhao Hu, Bo Liu, Li Wen, Mingfu Gong, Guangxian Wang, Li Yang, Yuyang Chen, Heng Chen, Hong Guo, Dong Zhang

**Affiliations:** ^1^Department of Radiology, XinQiao Hosptial, Third Military Medical University, ChongQing, China; ^2^Department of Gastroenterology, XinQiao Hosptial, Third Military Medical University, ChongQing, China

**Keywords:** ulcerative colitis, cognition, emotion, brain-gut axis, functional magnetic resonance imaging, amplitude of low-frequency fluctuation, functional connectivity

## Abstract

**Background**: Patients with ulcerative colitis (UC) usually display cognitive impairments, such as memory loss, attention deficits, and declining executive functions, particularly during the active stage of the disease. However, the potential neurological mechanisms of these symptoms remain unclear.

**Method**: Forty-one patients with mildly to moderately active UC, as well as 42 matched healthy controls, were recruited for an examination using psychological scales, cognitive function tests and resting-state functional magnetic resonance imaging (rs-fMRI). Seed points were identified *via* analysis of amplitude of low-frequency fluctuation (ALFF), and functional connectivity (FC) was calculated between these seed regions and other voxels in the whole brain. Correlation analyses were performed among clinical indexes, neuropsychological assessments and neuroimaging data.

**Result**: Compared with the healthy controls, patients with UC exhibited lower ALFF values in the bilateral hippocampal/parahippocampal (HIPP/ParaHIPP) region and higher ALFF values in the left posterior cingulate cortex (PCC.L) and left middle frontal gyrus (MFG.L). With HIPP/ParaHIPP as the seed point, the strengths of the FC in the bilateral middle frontal gyri (MFG), anterior cingulate cortex (ACC), and left caudate nucleus (CAU.L) increased; using the PCC.L as the seed point, the strengths of the FC in the middle cingulate cortex (MCC) and the left angular gyrus (AUG.L) increased. These abnormal brain regions were mainly located in the limbic system. By analyzing the correlations between these brain regions and behavioral data, we observed a close correlation between decreased HIPP/ParaHIPP activity and memory loss; increased PCC activity and strength of FC with the AUG.L were related to dysfunction of executive function and attention network in patients with UC.

**Conclusion**: Based on these results, the limbic lobe might be the core of the brain-gut axis (BGA) and play an important role in cognitive impairments, suggesting potential mechanisms for cognitive impairment in patients with UC in the active stage of the disease.

## Introduction

Ulcerative colitis (UC), a kind of inflammatory bowel disease (IBD), is an idiopathic, chronic inflammatory state of the colonic mucosa that is often characterized by bloody diarrhea (Ordã et al., 2012). Recovery is often difficult, with alternating periods of remission and activity that seriously affect the patient’s psychology and cognitive function (Han et al., 2010). Almost all patients with UC suffer from neuropsychiatric problems, including anxiety, depression, inattention, and insomnia. Cognitive impairment may also be more obvious and prominent in patients with UC during the active stage of the disease. However, the mechanisms underlying these symptoms in patients with UC remain to be explored.

Gut inflammation and brain cognitive function in brain may be connected neurologically. The brain-gut axis (BGA) hypothesis offers one explanation for the correlation between biological behavior, emotions and cognitive functions in subjects with UC (Mayer et al., 2006). According to the study by Agostini, the dysfunction of the amygdala in the BGA influences emotional processing in patients with UC during remission (Agostini et al., 2011). As shown in the study by JS Huang, the BGA may also play a central role in the perception of somatic pain stimuli in the brains of patients with IBD (Huang et al., 2016). BGA function has also been observed in many neuroimaging studies of patients with Crohn’s disease (Bao et al., 2016; Agostini et al., 2017). However, further studies are needed to determine whether BGA-related functional areas are abnormally activated in the brains of patients with active UC during the resting state. Furthermore, the correlation between cognitive impairment and the BGA in patients with UC remains unclear.

In the present study, we used resting-state functional magnetic resonance imaging (rs-fMRI) to investigate the spontaneous neural activity and functional connectivity (FC) in the brain of patients with UC. The amplitude of low-frequency fluctuation (ALFF) was used to detect alterations in regional brain activity. The ALFF reflects spontaneous neural activity in the cerebral region (Zhou et al., 2010) and has been widely employed to explore the latent mechanism of psychosomatic diseases, such as schizophrenia, insomnia, and Parkinson’s disease (Zhou et al., 2017). Whole-brain FC analyses were used to explore aberrant activity across brain regions, and time-related analysis of covariation with seed point ALFF was performed to identify peak points of brain regions as seeds for FC analyses (Biswal et al., 2010). The FC strength between the seed point and the altered brain area was used to identify the latent mechanisms of neurocognitive impairments in patients with UC. We hypothesized that regional and network-level brain functions associated with the BGA of patients with mildly to moderately active UC were altered and that the changes correlated with the results of neuropsychiatric evaluations.

## Materials and Methods

### Subjects

The study protocol was approved by the local Medical Research Ethics Committee of Xinqiao Hospital (Chongqing, China), and the study was conducted in accordance with the approved guidelines and regulations. Forty-one right-handed patients with UC (age range: 20–54 years, mean age: 37.1 ± 9.1 years, 16 females) were recruited from the IBD Clinic Center of Xinqiao Hospital and provided written informed consent after receiving a detailed description of the study procedures and aims.

Inclusion criteria were: (1) age 18–55 years; (2) education ≥6 school years; (3) mildly to moderately active UC (Mayo scores: 3–10; Lewis et al., 2008); and (4) right-handedness.

Exclusion criteria were: (1) clinical remission (Mayo scores <3) or severe UC [these patients were excluded because they usually need to take corticosteroids and accurate results from neuropsychological tests are difficult to obtain (Sands et al., 2010)]; (2) use of corticosteroids and psychotropic drugs in the past 6 months; (3) UC-related abdominal surgery; (4) previous or present neuro-associated diseases, such as psychosis, head tumor, or disturbance of consciousness; (5) claustrophobia; and (6) metallic implants present in the body.

Forty-two right-handed healthy controls (HCs; age range: 22–55 years, mean age: 36.86 ± 9.02 years, 17 females) were included in this study. Controls were matched with patients for age, sex, and education and were recruited from Xinqiao Hospital (Chongqing, China) using advertisements. None of the subjects in the HC group were taking any medication, had gastrointestinal or pain-related diseases, or tested positive in the colonoscopy. Similar to the UC group, MRI data from participants in the HC group whose maximum head motion exceeded 2.0 mm or 2.0° were rejected.

### Experimental Design

All patients underwent a systemic gastrointestinal examination, including a colonoscopy and pathological examination of a biopsy. The colonoscopies were completed in a 4-week period, and laboratory tests were performed within the 2 weeks before MRI. C-reactive protein (CRP) levels, erythrocyte sedimentation rate (ESR), platelet (PLT) counts, and Mayo scores were recorded for all patients. All participants performed neuropsychological assessments and cognitive function testing; before these tests, we ensured that subjects completely understood the rules. After explaining the test, we performed MRI scans on the subjects. MRI data were acquired during an approximately 10-min resting-state period.

### Clinical Symptoms Assessment and Neuropsychological Testing

Each subject underwent a series of related psychometrical assessments and clinical scales. Cognitive function tests were performed using the E-prime program. The mood tests included the Hospital Anxiety and Depression Scale (HADS; Hinz and Brähler, 2011), Self-rating Depression Scale (SDS, a 20-question self-reporting inventory designed to evaluate levels of depressive symptoms) and Self-rating Anxiety Scale (SAS, a 20-item self-reporting assessment device designed to measure anxiety levels). Clinical symptoms were mainly measured using the Inflammatory Bowel Disease Questionnaire (IBDQ, a questionnaire used to evaluate patient quality of life; Ren et al., 2010), the Pittsburgh Sleep Quality Index (PSQI), the Visual Analog Scale (VAS) and the Perceived Stress Scale (PSS).

We used three measures to evaluate cognition in each subject, including attention, executive function, and working memory. The Attention Network Task (ANT) provides efficiency measures of the alerting, orienting, and executive control networks (ECNs; Fossella et al., 2002; Fan et al., 2006). The Stroop Color-Word Test was used to evaluate executive function, and the memory of each subject was evaluated using a two-back working memory task. All three tests were performed in the E-prime program. During the tests, the accuracy of the answers and the mean reaction times (RTs) were recorded to reflect the test results.

### Image Data Acquisition

Each subject underwent rs-fMRI scanning on a 3.0-T GE MRI system equipped with a standard eight-channel phased-array head coil. During scanning, all patients were ordered to keep their eyes closed and to not fall asleep, remain equanimous, and have no systematic cognitive or motor activity. The echo planar imaging (EPI) sequence was applied to collect resting-state functional images using the following parameters: slices = 34; slice thickness = 5 mm, slice gap = 0 mm; TE = 30 ms, TR = 2,300 ms, flip angle (FA) = 90°, field of view (FOV) = 240 × 240 mm^2^, matrix = 64 × 64 and isotropic voxel size = 3 × 3 × 3 mm^3^. We collected 270 time points for approximately 621 s from each subject. A set of high-resolution T1-weighted structural images was collected by applying a three-dimensional fast spoiled gradient-echo (3DSPGR) sequence with the following parameters: slices = 124; slice thickness = 1.6 mm, slice gap = 0 mm; TE = 2.8 ms, TR = 450 ms, FA = 15°, FOV = 240 × 240 mm^2^, matrix = 256 × 256 and isotropic voxel size = 1.6 × 1.6 × 1.6 mm^3^.

### Image Data Preprocessing

MRI data were preprocessed using the Data Processing Assistant for Resting-State fMRI Advanced Edition^1^, which is based on MATLAB 8.2.0.701 (R2013b). Because of the time required for magnetization equilibrium and participant adjustment to the new and noisy environment, the first 10 volumes of each functional image were deleted. The remaining 260 functional images were slice-time corrected to reduce the differences in images from different times and realigned for head motion. Subjects whose maximum head motion was beyond 2.0 mm in any direction or whose maximum head rotation was beyond 2.0° in any angular dimension were excluded. The high-resolution T1-weighted structural images were then coregistered with the functional images. Next, the whole brain was segmented into gray matter (GM), white matter (WM) and cerebrospinal fluid (CSF). Subsequently, the regression of nuisance covariates was performed, including Friston 24-parameter correction and head motion scrubbing with thresholds of approximately 0.5 mm. Meanwhile, noisy signals from the CSF and WM were also regressed. Retaining or removing the global brain signals is controversial; we chose to retain the global signals. Then, all the data were normalized into the standard Montreal Neurological Institute (MNI) template, and each voxel size was resampled at 3 × 3 × 3 mm and filtered at the 0.01–0.08 Hz band. Finally, we set a Gaussian kernel of 6-mm full-width at half-maximum (FWHM) to conduct spatial smoothing. In addition, detrending was used to remove the linear trends.

The whole-brain ALFF was calculated for each subject using the preprocessed images with temporal bandpass filtering (0.01 < *f* < 0.08 Hz), which reduces low-frequency drift and high-frequency respiratory and cardiac noise. The subject-level voxelwise ALFF map was converted into a z-score map by subtracting the mean ALFF of the whole brain and dividing by the standard deviation (Li et al., 2012). FC was calculated in MATLAB using the rs-fMRI data analysis toolkit (REST v1.8)^2^. The peak points of the ALFF analysis were selected as the coordinates of those regions of interest (ROIs), and the radius was set as 6 mm. Seed-based FC maps were calculated between the time courses of seed regions and the time series of all voxels in the global brain by Pearson’s correlation analyses. Finally, Fisher’s r-to-z transformation was applied to all maps of before statistical analysis.

### Statistical Analysis

Statistical analyses were performed using SPSS 22.0 (SPSS Inc., Chicago, IL, USA). Independent sample *t*-tests were used to analyze differences in the clinical and neuropsychological data between the two groups (41 patients and 42 HCs). Additionally, if the variable did not exhibit a normal distribution, the Mann-Whitney rank test was used for analysis. The chi-square test was used to detect differences in sex, which was the categorical variable. All *p* values were two-sided, and statistical significance was set to *p* < 0.05.

We investigated differences in ALFF and FC in the 41 patients with UC compared with the 42 HCs. Analysis of the ALFF and FC maps were performed with SPM8^3^. Age, sex, and educational level were used as covariates during the two-sample *t*-test calculation. First, we used the topological false discovery rate (FDR) to correct for multiple comparisons (Chumbley et al., 2010), which is a rigid approach for spm maps. For all of the aforementioned analyses, we set a voxel-level threshold of *p* = 0.001 and an FDR statistical significance of *p* < 0.05; the FDR correction was applied to multiple comparisons in the whole brain. Meanwhile, a GM group mask was utilized in the ALFF and FC calculations. Second, small volume correction (SVC) was applied over the following cerebral regions: frontal gyrus, hippocampus, caudate nucleus, and cingulate cortex. In previous studies, these brain regions were aberrantly altered in patients with UC (Agostini et al., 2011; Gray et al., 2018). SVC was conducted by applying a familywise error (FWE) correction of *p* < 0.05 and a cluster size threshold of 20 contiguous voxels in the brain region (Vachon et al., 2013). Compared with FDR correction, SVC correction is a hypothesis-driven analysis approach for multiple comparisons, specifically within ROIs instead of using other stricter corrections for the whole brain (Torres et al., 2016).

Pearson’s correlation coefficients were calculated to assess relationships among clinical measures, neuropsychological results, ALFF values, and the intensity of FC in patients with UC. Calculations were performed using SPSS software after eliminating the influences of age, sex, and education, and statistical significance was set to *p* < 0.05.

## Results

### Clinical Characteristics and Neuropsychological Results

The results of the statistical analysis of demographic, clinical, and neuropsychological characteristics between patients with UC and HCs are shown in Table 1. The demographics, including age, sex, and education level, were not significantly different between the two groups. Compared with the HC group, patients with UC showed prominently lower scores on the IBDQ. The PSQI, VAS, and PSS scores of the UC group were significantly higher than those of the HC group. The HADS, SAS, and SDS scores were also significantly different between the two groups. Patients with UC scored higher on the emotion scales than the HC group. Additionally, the results of the neuropsychological assessments showed that the UC group had a longer alerting time, executive time, two-back working memory RT, and Stroop test RT but a lower accuracy rating in the two-back working memory and Stroop tests. The two groups did not differ significantly in the orienting effect time.

**Table 1:** Demographics, clinical data, mood and cognitive performance among Ulcerative colitis (UC) and healthy control (HC).

Characteristic	UC patients (*n* = 41)	Heathy controls (*n* = 42)	*p* Value
Age (years)	37.10 ± 9.45	36.86 ± 9.02	0.766^a^
Gender (male/female)	25/16	25/17	0.943^b^
Education (years)	11.85 ± 3.62	12.48 ± 3.20	0.383^a^
Disease duration (months)	39.58 ± 39.28		
Montreal classification	E1:E2:E3 = 11:12:18		
Mayo	4.66 ± 1.53		
CRP	8.58 ± 7.91		
ESR	12.67 ± 10.02		
PLT	289.00 ± 94.87		
IBDQ	152.07 ± 32.28	193.04 ± 17.32	<0.001^a^
PSQI	8.48 ± 4.53	3.40 ± 2.25	<0.001^a^
VAS	1 (0–7)	0 (0–2)	<0.001^c^
PSS	17.95 ± 9.27	14.88 ± 7.02	0.013^a^
**Emotion**			
HADS-A	6.29 ± 3.43	4.45 ± 2.43	0.016^a^
HADS-D	5.78 ± 3.97	4.09 ± 2.27	<0.001^a^
SAS	43.78 ± 11.17	32.05 ± 5.64	<0.001^a^
SDS	39.32 ± 10.19	33.54 ± 7.23	0.002^a^
**Attention**			
ANT alerting effect (ms)	45.63 ± 26.04	36.50 ± 15.75	0.048^a^
ANT orienting effect (ms)	25.51 ± 34.05	29.76 ± 33.62	0.806^a^
ANT executive effect (ms)	115.07 ± 43.36	92.98 ± 34.58	0.044^a^
**Executive function**			
ACC of stroop (%)	87.0 (69.2–95.0)	95.0 (88.0–97.0)	<0.001^c^
Stroop RT (ms)	791.52 ± 119.92	663.49 ± 81.72	0.022^a^
**Work memory**			
ACC of two-back (%)	73.0 (65.5–82.0)	87.0 (78.0–91.25)	<0.001^c^
Two-back RT (ms)	722.28 ± 137.86	638.30 ± 97.54	0.031^a^
**Medication**			
Mesalazine SR granules (3 g, po, qd)	E2 + E3 (30) 30 patients		
Mesalazine suppository (1 g, rectally, qn)	E1 (11) 11 patients		

*Abbreviations: CPR, C-reactive protein; ESR, erythrocyte sedimentation rate; PLT, platelet count; IBDQ, Inflammatory Bowel Disease Questionnaire; HADS, Hospital Anxiety and Depression Scale; SAS, Self-Rating Anxiety Scale; SDS, Self-Rating Depression Scale; PSQI, Pittsburgh Sleep Quality Index; VAS, Visual Analogue Scale; ANT, Attention Network Task; ACC, accuracy rating; RT, reaction time. ^a^Two-sample independent *t*-test. ^b^Chi-square test. ^*c*^Mann-Whitney rank test*.

### ALFF Analysis

**Figure 1:**
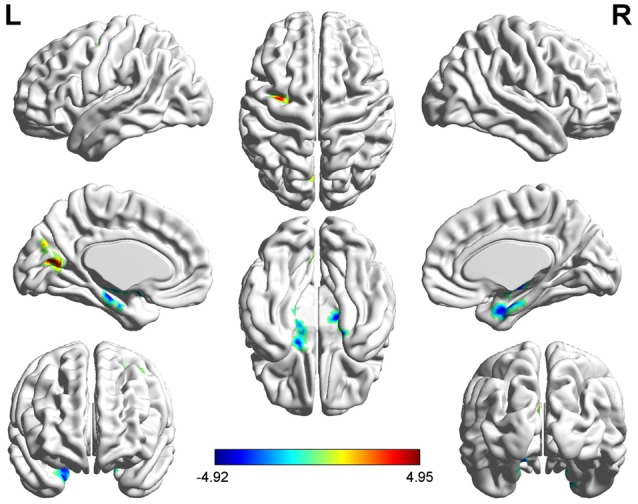
Significantly increased (red) and decreased (blue) amplitude of low-frequency fluctuation (ALFF) values in patients with active-stage ulcerative colitis (UC) compared with healthy controls [HCs; peak level threshold *p* = 0.001, cluster level false discovery rate (FDR)-corrected *p* < 0.05]. The color bar represents the *t*-value of the two-sample *t*-test between the two groups.

Several related brain regions showed significant ALFF alterations in patients with UC compared with HCs (FDR-corrected, cluster level *p* < 0.05 and voxel-level threshold of *p* = 0.001; Figure 1). Patients with UC displayed increased ALFF values, with the peak differences in the left middle frontal gyrus (MFG.L) and the left posterior cingulate cortex (PCC.L), and decreased ALFF values in the bilateral hippocampus/parahippocampus (HIPP/ParaHIPP). We select the aforementioned peak points as seeds for further FC analyses. The results are summarized in Table 2.

**Table 2:** ALFF alterations between the UC and HC groups.

			MNI peak point coordinates				
Brain region	BA	Hem	*X*	*Y*	*Z*	*t*-Value	Voxels
HIPP/ParaHIPP	35	L&R	12	−3	−30	−4.9212	253
PCC	31	L	−15	−66	−12	4.3442	58
MFG	9/10	L	−18	−6	57	4.9545	51

*Notes: MNI, Montreal Neurological Institute; ALFF, amplitude of low-frequency fluctuations; BA, Brodmann area; Hem, hemisphere; HIPP/ParaHIPP, hippocampus/parahippocampus; PCC, posterior cingulate cortex; MFG, middle frontal gyri; false discovery rate (FDR)-corrected, p < 0.05*.

### FC Analysis

Three peak points, including the MFG.L, PCC.L and HIPP/ParaHIPP, were set as seed points; two-sample *t*-tests were performed in SPM8 to identify significantly altered FC between seed points with the whole brain (Table 3).

**Table 3:** FC alterations between the UC and HC groups.

				MNI coordinates				
Connected regions	BA	Hem	Peak areas	*X*	*Y*	*Z*	*t*-Value	Voxels
Seed point (12, −3, −30)								
	10	L&R	MFG^a^	18	54	6	4.8661	209
	24	R	ACC^a^	21	30	−12	4.0674	184
	−	L	CAU^b^	−12	12	−6	3.938	57
Seed point (−15, −66, 12)								
	31	L&R	MCC^a^	0	−30	36	4.7667	562
	39	L	ANG^a^	−33	−63	30	4.2585	153
	19	R	IOG^a^	30	−87	−9	−4.137	74
Seed point (−18, −6, 57)								
	−	−	Brainstem^a^	9	−9	−18	5.7835	125
	40	R	IPL^a^	39	−48	42	4.6097	110
	6	R	SFG^a^	21	0	51	4.4795	99

*Notes: FC, functional connectivity; MFG, middle frontal gyri; ACC, anterior cingulate cortex; CAU, caudate nucleus; MCC, middle cingulate cortex; ANG, angular gyrus; IOG, inferior occipital gyrus; IPL, inferior parietal lobule; SFG, superior frontal gyrus. ^a^FDR-corrected *p* < 0.05. ^b^Small volume-corrected (FWE-corrected at *p* < 0.05) with a cluster size threshold of 20 contiguous voxels*.

**Figure 2:**
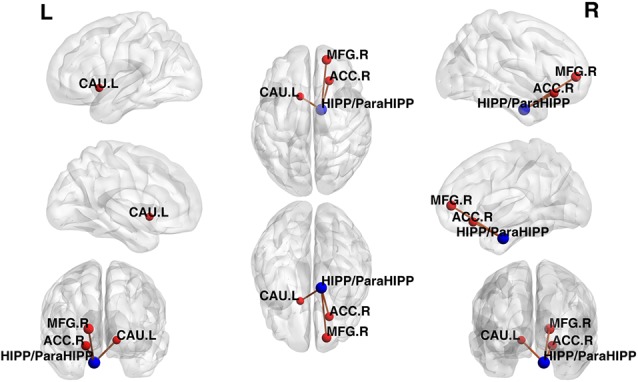
Differences in functional connectivity (FC) between various brain regions between patients with active-stage UC and HCs. The peak level threshold was set to *p* = 0.001, and the cluster level FDR-corrected threshold was set to *p* < 0.05. The FC between the seed point and left caudate nucleus (CAU.L) used the small volume correction (SVC)-corrected *p* value familywise error (FWE-corrected *p* < 0.05). The blue node was the seed point and all the FC strengths were increased.

Using the HIPP/ParaHIPP, whose peak point was (Postle and D’Esposito, 1999; Mayer et al., 2006; Sands et al., 2010), as the first seed point, a significantly increased FC strength was revealed in some brain regions, including the bilateral middle frontal gyri (MFG), the anterior cingulate cortex (ACC), and the left caudate nucleus (CAU.L; *p* < 0.05, FDR-corrected or SVC-corrected; Figure 2).

**Figure 3:**
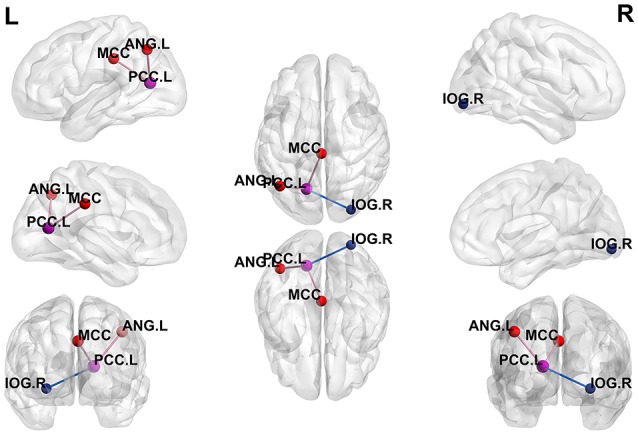
Differences in whole-brain FC between patients with active-stage UC and HCs. The peak level threshold was set to *p* = 0.001, and the cluster level FDR-corrected threshold was set to *p* < 0.05. The purple node represents the seed point, the purple lines indicate an increased FC strength and the blue line indicates a decreased FC strength.

Using the peak point (−15, −66, 12) of the PCC.L as the second seed point, significantly increased FC strength was observed in the middle cingulate cortex (MCC) and the left angular gyrus (ANG.L). In addition, decreased FC strength was revealed in the right inferior occipital gyrus (IOG.R; *p* < 0.05, FDR-corrected; Figure 3).

**Figure 4:**
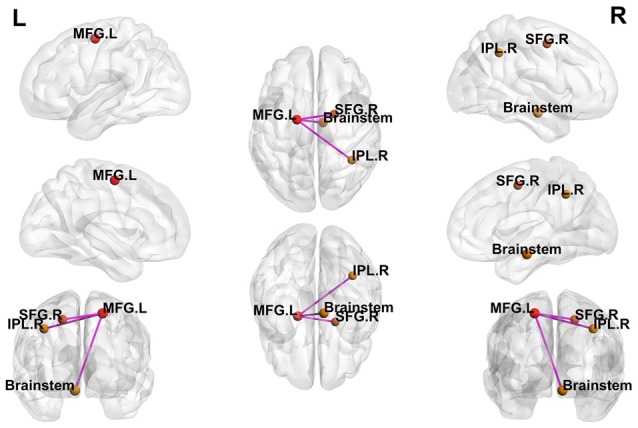
Differences in whole-brain FC between patients with active-stage UC and HCs. The peak level threshold was set to *p* = 0.001, and the cluster level FDR-corrected threshold was set to *p* < 0.05. The purple node represents the seed point; all FC strengths were increased.

Using the peak point (−18, −6, 57) of the MFG.L as the third seed point, increased FC strength was revealed in some brain regions, including the brainstem, right inferior parietal lobule (IPL.R), and right superior frontal gyrus (SFG.R; *p* < 0.05, FDR-corrected; Figure 4).

### Correlation Among Clinical Data, Neuropsychological Data, and Aberrant ALFF and FC Values

**Figure 5:**
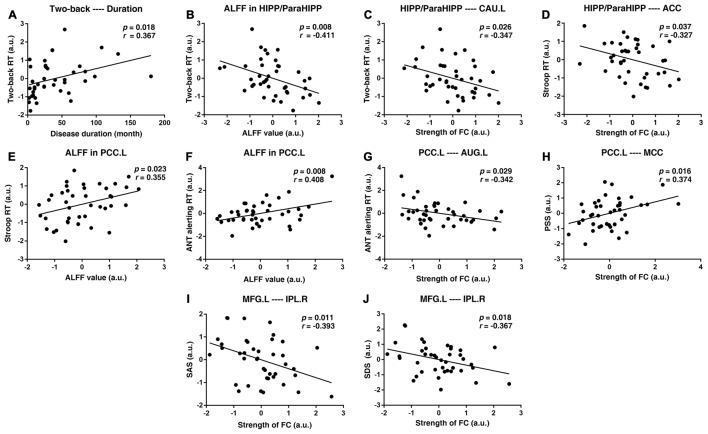
Scatter diagrams showing the significant correlations between aberrant ALFF values, FC strengths, clinical data, and neuropsychological assessments of patients with UC. **(A)** Disease duration was positively correlated with the two-back reaction time (RT).** (B,C)** The two-back RT was negatively correlated with the decreased ALFF value of the HIPP/ParaHIPP and negatively correlated with increased FC strength between the HIPP/ParaHIPP and CAU.L. **(D,E)** The RT of the Stroop test was positively correlated with the increased ALFF value of the left posterior cingulate cortex (PCC.L) and negatively correlated with increased FC strength between the HIPP/ParaHIPP and anterior cingulate cortex (ACC). **(F,G)** The alerting effect of the attention network task (ANT) was positively correlated with the ALFF value of the PCC and negatively correlated with increased FC strength between the PCC.L and AUG.L. **(H)** The perceived stress scale (PSS) score was positively correlated with increased FC strength between the PCC.L and middle cingulate cortex (MCC). **(I,J)** The increased FC strength between the MFG.L and IPL.R was negatively correlated with the self-rating anxiety scale (SAS) and self-rating depression scale (SDS) scores.

After controlling for age, sex, and educational level, we performed a partial correlation analysis of the UC group. First, the disease duration was positively correlated with the two-back RT (*p =* 0.018*, r =* 0.367). Subsequently, RT in the two-back working memory test was negatively correlated with the decreased ALFF value of the HIPP/ParaHIPP and negatively correlated with the increased FC strength between the HIPP/ParaHIPP and CAU.L (*p* = 0.008, *r* = −0.411; *p* = 0.026, *r* = −0.347, respectively). Moreover, the RT of the Stroop test was positively correlated with the increased ALFF value of the PCC.L and negatively correlated with increased FC strength between the HIPP/ParaHIPP and ACC (*p* = 0.023, *r* = 0.355; *p* = 0.037, *r* = −0.327, respectively). Additionally, the alerting effect of the ANT was positively correlated with the ALFF value of PCC.L and negatively correlated with the increased FC strength between the PCC.L and AUG.L (*p* = 0.008, *r* = 0.408; *p* = 0.029, *r* = −0.342, respectively). Besides, the PSS was positively correlated with the increased FC strength between PCC.L and MCC (*p* = 0.016, *r* = 0.374). Finally, the increased FC strength between the MFG.L and IPL.R was negatively correlated with the SDS and SAS scores (*p* = 0.018, *r* = −0.367; *p* = 0.011, *r* = −0.393, respectively). These correlation analyses are illustrated in Figure 5.

As a supplementary approach, we also extracted brain function signals from the same brain regions in the HC group as in patients displaying changes in brain function and analyzed the correlation between these values and their behavioral data. However, no significant brain-behavior correlations were identified in the HC group.

## Discussion

This rs-fMRI study investigated aberrant brain activity in patients with mildly to moderately active UC and speculated on the neural mechanism underlying their cognitive impairment. In contrast to previous studies, our work not only revealed regional brain activity in the resting state but also analyzed the seed-based whole-brain FC to detect abnormal brain functions in patients with UC. As we predicted, our findings revealed alterations in the limbic system, including the bilateral HIPP/ParaHIPP and left PCC, through the ALFF analysis. In addition, further seed-based FC analyses identified additional aberrantly activated regions that are involved in the default mode network (DMN; Jolles et al., 2013), salience network (SN) and ECN (Ng et al., 2016). Analyses of psychological scales and cognitive function evaluations revealed that both emotional and cognitive functions were impaired in patients with UC compared with HCs. Notably, correlation analyses indicated that abnormal activity in some regions of the brain was related to cognitive function and emotion in patients with UC, and no significant brain-behavior correlations were observed in the HCs. Thus, these correlations appeared only in the UC group. As discussed below, our results illuminate the potential mechanisms underlying cognitive impairments and neuropsychological complications in patients with UC.

The altered ALFF values in brain regions primarily located in the limbic system were related to the BGA. The gut communicates information to the central nervous system (CNS) through the enteric nervous system (ENS), including the sympathetic and parasympathetic nervous systems. The CNS responds to and affects intestinal homeostasis by communicating with the ENS, and the BGA plays a crucial role in this communication (Huang et al., 2016). According to the BGA hypothesis, limbic brain regions such as the medial-temporal and cingulate cortices have a direct link to the intrinsic neural network of the gastrointestinal tract (Agostini et al., 2011). Abnormal activity in the BGA can lead not only to increased intestinal inflammation but also to changes in brain function, especially cognitive function. In animal studies, rat forebrain regions including the cortex, hypothalamus, and limbic areas have been suggested to comprise the BGA (Tsurugizawa et al., 2009). Recent fMRI studies revealed abnormalities in the hippocampus or parahippocampal region, amygdala, caudate, and frontal regions in patients with IBD (Agostini et al., 2017), suggesting that these brain areas might contribute to the BGA. In the present study, patients with UC experienced constant intestinal inflammation, and their memory capabilities declined to varying degrees. As the disease duration increased, patient RT in the two-back working memory test increased. The two-back RT also exhibited negatively correlated with subdued ALFF values in HIPP/ParaHIPP, but notably, the FC strength between the HIPP/ParaHIPP and CAU.L increased. The hippocampus plays an important role in the formation of new memories of experienced events, as confirmed by studies using hippocampal cell transplantation in primates (Virley et al., 1999). In addition, the parahippocampal gyrus functions in the encoding and recognition of environmental scenes (Mégevand et al., 2014). Patients with UC showed decreased HIPP/ParaHIPP activity and longer RT of two-back test, suggesting that their impairments in working memory were associated with decreased HIPP/ParaHIPP activity. The caudate nucleus is involved in the dorsal-prefrontal cortex-subcortical loop, which correlates with deficits in working memory (McHaffie et al., 2005). Functional imaging has revealed activation of this subcortical loop during working memory tasks in primates and healthy human subjects (Hannan et al., 2010). The caudate may also be associated with deficits involving working memory before disease onset. A voxel-based morphometry (VBM) study reported a correlation between the volume of the caudate nucleus and perseverative errors in spatial working memory tasks (Levitt et al., 2002). The amygdala sends direct projections to the caudate nucleus, and both regions have direct and indirect projections to the hippocampus (Postle and D’Esposito, 1999). The increased FC strength between the HIPP/ParaHIPP and CAU.L might be a compensatory mechanism of the human brain to reduce the effect of decreased hippocampal activity on working memory. HIPP/ParaHIPP function and FC with CAU.L are postulated to be related to memory deficits in patients with UC.

The cingulate cortex also plays a central role in the BGA and can be activated by inflammatory stimuli from the gut to affect emotional and cognitive functions in the brain (Elsenbruch et al., 2010). Different regions of the cingulate cortex from neural networks responsible for different functions, including encoding emotions, motivations and cognitive demands (Price, 2000; Davis et al., 2005). First, the ACC is primarily responsible for interoceptive processing in the brain (Craig, 2002), and a study employing the Stroop test confirmed that the ACC is involved in error detection and conflict monitoring (Bush et al., 2000). In the present study, the FC strength between the HIPP/ParaHIPP and ACC was increased and negatively correlated with RT in the Stroop test. This network might also be a self-preservation mechanism of the brain to counteract impaired executive function. Second, the PCC is highly connected and one of the most metabolically active regions in the brain, having been characterized as a central node in the DMN regulating cognitive and mental function (Andrewshanna et al., 2014). The PCC also participates in the dorsal attention network, which integrates multimodal information and is involved in attentional processes and memory formation (Leech and Sharp, 2014). The PCC switches brain activity between introspective functions of the DMN and externally focused functions of the executive network (Menon and Uddin, 2010). Previous neuroimaging studies have identified positive PCC activation in IBD patients (Rubio et al., 2016). In our research, the ALFF value of the PCC increased and was positively correlated with the alerting effect of the ANT and RT of the Stroop test. This observation further indicates that the abnormal activity of the PCC results in impaired attention and executive function in patients with UC. Moreover, the FC strength between the PCC.L and AUG.L was increased and negatively correlated with the alerting effect of the ANT. The ANG is also part of the DMN and is associated with attention to visual-spatial features (Seghier, 2013). This region plays a decisive role in distinguishing left or right by understanding conceptual “left” or “right” based on spatial position (Hirnstein et al., 2011). Notably, the ANT task involves determining the right and left directions of the arrow to assess attention in patients with UC. Based on our findings, the ANG plays an important role in the compensatory mechanism of attention processing. When patients with UC show impaired attention, increased FC with the PCC enhances the alerting effect of ANT. Third, the MCC forms extensive connections with the amygdala and participates in the stress and emotional regulation of pain (Vogt, 2005). Under stress conditions, aberrantly increased MCC activity might amplify visceral sensitivity in patients with IBD (Agostini et al., 2017). In the present study, the FC between the PCC.L and MCC increased and positively correlated with the PSS score. Thus, the MCC might be the foundation of perceived stress in patients with UC. In summary, the cingulate cortex, which is part of the limbic system, plays an important role in the BGA and affects the executive function and attention of patients with UC.

Finally, the ALFF value of the MFG.L increased and enhanced the FC strength from the MFG.L to the IPL.R and SFG.R. The frontal lobe is associated with emotional modification, receiving input from the limbic system and playing an important part in the storage of emotional memory (Bermpohl et al., 2010). The prefrontal lobe was recently shown to be involved in attention to negative or positive emotions and is always abnormal in patients with IBD (Kerestes et al., 2012). When people participate in negative-based emotion learning, the medial prefrontal gyri and the ACC are strongly activated, and abnormalities in the emotion circuit may lead to symptoms of anxiety and depression (Lin et al., 2012). A resting-state frontal electroencephalograph (EEG) study revealed an association between abnormal asymmetry of frontal lobe electroencephalograph (EEG) and emotion-related disturbances, such as anxiety and depression (Thibodeau et al., 2006). The development of frontal lobe function in children is very important for emotional intelligence (Rosso et al., 2010). In the current study, the SAS and SDS scores of patients with UC were higher than those of the HCs and negatively correlated with increased FC strength from the MFG.L to the IPL.R. We speculate that this increase in FC might be responsible for the susceptibility of patients with UC to developing anxiety and depression. The strength of FC from the MFG.L to the IPL.R may be involved in the emotion circuit of patients with UC. However, further research is needed to confirm this mechanism.

This work also has several limitations. First, this study found aberrant brain activity in patients with mildly to moderately active UC, but further investigations are needed to determine whether this regional abnormal brain activity is reversible during remission in patients with UC. Second, we sought to eliminate all uncertainties and create a perfect experimental environment; however, some patients who suffered from abdominal discomfort showed poor coordination in the cognitive function evaluation. Finally, social experience, including profession or work-related stress, was another uncontrollable factor that might have contributed to the observations of aberrant brain function. Considering these limitations, the above factors should be considered in subsequent studies, and the changes in brain activity between patients with clinical remission and active UC should be further explored.

## Conclusion

In conclusion, the current study observed dysfunction in the regional and network-level brain functions in patients with mildly to moderately active UC using rs-fMRI. The limbic system, including the hippocampus, parahippocampal gyrus, and cingulate cortex, may be considered the center of the BGA, which bidirectionally influences cognitive and gastrointestinal functions. In addition, the DMN and the aberrant FC involving the caudate nucleus, ANG, and MFG may also be a potential neural circuit related to cognitive impairments in patients with UC. These findings provide a better understanding of BGA function, as well as strong neuroimaging evidence of brain dysfunction in patients with UC.

## Data Availability

The datasets for this manuscript are not publicly available because UC patients’ MRI date the privacy of them and is not available to the public. Requests to access the datasets should be directed to zhangdongxqyy@163.com.

## Author Contributions

WF contributed to the experiments, data analysis and writing of the manuscript, and as the first author. SZ and BL contributed to performing the experiments and writing and revising the manuscript. LW designed the experiment and revised the manuscript. JH, MG and GW contributed to the data analysis and manuscript revision. LY, HC and YC contributed to the collection of patients. DZ and HG are the guarantors of this study and had complete access to all data in the study, and contributed equally to this work as the corresponding authors. They accept responsibility for the integrity of the data and the accuracy of the data analysis.

## Conflict of Interest Statement

The authors declare that the research was conducted in the absence of any commercial or financial relationships that could be construed as a potential conflict of interest.

The reviewer XY declared a shared affiliation, though no other collaboration, with the authors to the handling Editor.

## Footnotes

^1^http://rfmri.org/dpabi

^2^http://www.restfmri.net/forum/REST_V1.8

^3^http://www.fil.ion.ucl.ac.uk/spm/
